# Facile Controlled Synthesis of Spinel LiMn_2_O_4_ Porous Microspheres as Cathode Material for Lithium Ion Batteries

**DOI:** 10.3389/fchem.2019.00437

**Published:** 2019-06-14

**Authors:** Yun Hai, Ziwei Zhang, Hao Liu, Libing Liao, Peng Fan, Yuanyuan Wu, Guocheng Lv, Lefu Mei

**Affiliations:** ^1^Beijing Key Laboratory of Materials Utilization of Nonmetallic Minerals and Solid Wastes, School of Materials Science and Technology, China University of Geosciences, Beijing, China; ^2^School of Science, China University of Geosciences, Beijing, China

**Keywords:** lithium manganese oxide, microsphere, cathode, lithium ion battery, percipitation, calcination

## Abstract

Although the electrochemical properties of porous LiMn_2_O_4_ microspheres are usually improved compared to those of irregular LiMn_2_O_4_ particles, the effects of the different synthesis conditions on the preparation of the porous LiMn_2_O_4_ microspheres are rarely discussed in detail. In the present work, porous LiMn_2_O_4_ microspheres were successfully synthesized by using molten LiOH and porous Mn_2_O_3_ spheres as a template. Multiple factors were considered in the preparation process, including reagent concentration, pH, adding mode, heating time, etc. The morphology of the MnCO_3_ template was crucial for the preparation of porous LiMn_2_O_4_ microspheres and it was mainly affected by the concentration of reactants and the pH value of the solution during the precipitation process. During the lithiation of Mn_2_O_3_ microspheres, the heating temperature and the ratio between Mn_2_O_3_ and lithium salt were the most significant variables in terms of control over the morphology and purity of the LiMn_2_O_4_ microspheres. Furthermore, we demonstrated that the porous LiMn_2_O_4_ microspheres presented better rate capability and cyclability compared to commercial LiMn_2_O_4_ powder as cathode materials for lithium-ion batteries (LIBs). This study not only highlights the shape-controllable synthesis of LiMn_2_O_4_ microspheres as promising cathode materials, but also provides some useful guidance for the synthesis of porous LiMn_2_O_4_ microspheres and other LIB' electrode materials.

## Introduction

With the development of science and technology, lithium-ion batteries (LIBs) have been widely used in various portable energy storage devices (Wakihara, 2001; Etacheri et al., 2011; Chen D. et al., 2018; Chen H. et al., 2018) such as tablets, smartphones, cameras, etc. Further applications in electric vehicles, hybrid vehicles, military, and aerospace have also been developed and explored (Smart et al., 2004; Park et al., 2010; Lu et al., 2013; Lipu et al., 2018). Among various cathode materials for LIBs, LiMn_2_O_4_ cathode material with a spinel structure—which is cheap, safe and rich in resources—has become a research hotspot (Lee et al., 2010; Qu et al., 2011; Lai et al., 2016; Zhu et al., 2016; Wang et al., 2018). In long-term charging and discharging, the dissolution of Mn into electrolytes causes capacity degradation and poor cycle performance of cathode materials, which severely restricts the commercial application of LiMn_2_O_4_. Various methods have been tried to improve the electrochemical performance of spinel LiMn_2_O_4_ cathode materials, including surface coating, bulk doping, and morphology control (Iqbal et al., 2012; Tang et al., 2013; Jeong et al., 2015; Xu et al., 2018). Among these improvements, down-sizing of LiMn_2_O_4_ particles can significantly shorten the transport distance of lithium ions in solid, thus helping to improve their rate performance. Therefore, nanostructured LiMn_2_O_4_ with various morphologies has been extensively prepared in recent years (Lee et al., 2010; Tang et al., 2012, 2013). Tang et al. (2012) synthesized a nanochain of LiMn_2_O_4_ by a sol-gel method with a very good rate capability. The result showed a reversible capacity of 100 mAhg^−1^ at rate of about 1 C and 58 mAh g^−1^ at rate of 20 C. Lee et al. (2010) synthesized ultrathin LiMn_2_O_4_ nanowires with diameters <10 nm and lengths of several micrometers, which displayed 100 and 78 mAh g^−1^ at very high rates of 60 and 150 C. Nevertheless, the tap density of the above nanostructured LiMn_2_O_4_ is generally low due to its irregular shape, high surface area, and high porosity (Guo et al., 2014), resulting in low volumetric energy density of the LIBs' electrodes. In contrast, the LIBs' electrodes made of sub-micron-sized spherical active materials usually show higher volumetric energy density (Aurbach et al., 1999; Levi et al., 1999; He et al., 2006), which is caused by the compact packing of spherical particles. Additionally, an ideal structure would be a porous microsphere which consists of nanocrystallites tightly compacted with three-dimensional channels for ion diffusion in consideration of electron transportation distance (Qian et al., 2010; Ren et al., 2014; Yin et al., 2019). This structured LiMn_2_O_4_ can have both high volumetric energy density and high rate capability simultaneously. For example, Liu et al. (2018) reported the synthesis of the porous LiMn_2_O_4_ micro-/nano-hollow spheres from the globe precursor MnCO_3_ via a facile precipitation route. The obtained LiMn_2_O_4_ delivered excellent cycle stability and almost no capacity loss after 200 cycles. Wang et al. (2013) synthesized porous LiMn_2_O_4_ spheres with pores at an average size of 45 nm. The discharge capacity of the porous sphere LiMn_2_O_4_ was 83 mAh g ^−1^ at a rate of 20 C, which showed stable high-rate capability. Although the previous work has reported the preparation of porous LiMn_2_O_4_ microspheres and improvement of their electrochemical properties, the effects of the different synthesis conditions on the LiMn_2_O_4_ morphology and size have been rarely discussed in detail.

Herein, we reported the synthesis of spinel LiMn_2_O_4_ porous microspheres by lithiation of porous Mn_2_O_3_ microspheres. The effects of a series of preparation conditions, including reagent concentration, pH, adding mode, heating time, etc., on the morphology of the LiMn_2_O_4_ microspheres were investigated in detail. Moreover, we compared the electrochemical performance of synthesized LiMn_2_O_4_ microspheres with that of commercial LiMn_2_O_4_ powder. Significantly, without cation doping or surface coating, the porous LiMn_2_O_4_ microspheres present better rate capability and cyclability.

## Experimental

### Materials Synthesis

#### Preparation of MnCO_3_ Microsphere

LiOH·H_2_O (99.0%, AR) and ethanol (99.7%, AR) were purchased from Aladdin. MnSO_4_·H_2_O (99.0%, AR), NH_4_HCO_3_ (21.0%, AR), NH_3_·H_2_O (25%, AR), and H_2_SO_4_ (98.0 wt.%) were supplied by XiLong Chemical Co. Ltd. All reagents were used without further purification. The spherical MnCO_3_ was first prepared by a general chemical precipitation method. In a typical synthesis, 0.3042 g MnSO_4_·H_2_O and 1.4231 g NH_4_HCO_3_ were dissolved in 45 mL deionized water and 5 mL ethanol, respectively, to form a transparent solution. After the complete dispersion of the MnSO_4_ and NH_4_HCO_3_ solutions, the NH_4_HCO_3_ solution was added to the MnSO_4_ solution rapidly with vigorous stirring. A certain amount of NH_3_·H_2_O (10.0% v/v.) or H_2_SO_4_ (10.0% v/v.) was then added dropwise to adjust the pH value of the suspension to 7.5. The milky white suspension was stirred for 3 h at room temperature and maintained for 5 h. The powder was obtained by filtrating, washing, and drying in the air at 80°C for 24 h to obtain spherical MnCO_3_ precursors.

#### Preparation of Mn_2_O_3_ and LiMn_2_O_4_

The as-obtained MnCO_3_ powders were heated in air at 700°C for 10 h at a heating rate of 10°C·min^−1^ to synthesize porous Mn_2_O_3_ spheres. The porous Mn_2_O_3_ spheres were grounded thoroughly with LiOH·H_2_O in a molar ratio of Mn_2_O_3_:LiOH = 1:1.1 using ethanol as a dispersal agent. Finally, the mixtures were calcined at 650°C for 10 h with a heating rate of 5°C·min^−1^ in the air to achieve porous LiMn_2_O_4_ spheres.

### Characterization

X-ray diffraction (XRD) characterization was conducted to identify the crystal structure of the samples on an X-ray powder diffractometer (D8 Advance, Bruker, Germany) with a Cu Kα (λ = 0.15406 Å) radiation. The micro-morphologies of MnCO_3_, Mn_2_O_3_, and LiMn_2_O_4_ were observed using scanning electron microscopy (FESEM, MERLIN VP Compact, ZEISS, Germany).

### Electrochemical Measurements

The electrochemical performance was evaluated using CR2032 coin cells assembled in a high-purity argon-filled glove box with the moisture and oxygen content maintained below 0.1 ppm. The working electrode consisted of 70 wt.% LiMn_2_O_4_ spheres, 20 wt.% acetylene black and 10 wt.% polyvinylidene fluoride (PVDF) as a binder. Pure lithium foil was used as the counter electrode. The separator was Celgard 2400. The electrolyte was 1.0 M LiPF_6_ in ethylene carbonate/ethyl methyl carbonate/dimethyl carbonate solvent (1:1:1 v/v/v, Shenzhen Keijing Star Tech. Co.). The cells were aged for 12 h before measurement. All cyclic voltammogram tests were performed on an electrochemical workstation (Ivium-Vertex, Ivium Technologies, Holland). The galvanostatic charge/discharge tests were carried out using a Battery Testing system (CT-4008, NEWARE, China) with a voltage window of 3.0–4.5 V vs. Li^+^/Li at room temperature.

## Results and Discussion

### The Synthesis Principle and Process of LiMn_2_O_4_ Microspheres

**Figure 1:**
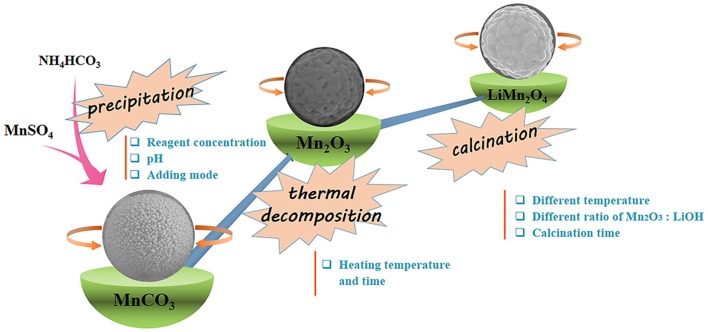
Schematic of the preparation of the spinel LiMn_2_O_4_ porous microspheres.

Figure 1 shows the schematic of the preparation of the spinel LiMn_2_O_4_ porous microspheres. First, MnCO_3_ microspheres as precursors are synthesized based on a precipitation method through the reaction of the MnSO_4_ solution with the NH_4_HCO_3_ solution. Secondly, MnCO_3_ microspheres are subsequently transformed into Mn_2_O_3_ microspheres through the decomposition by heating treatment in tube furnace. Finally, LiMn_2_O_4_ microspheres are obtained by the lithiation reaction of Mn_2_O_3_ porous microspheres with LiOH at high temperature. The influences of the key parameters in each of the above processes on the microstructures and crystallinities of the products are further discussed in detail.

### The Effects of Main Preparation Conditions on the Morphology of MnCO_3_

**Figure 2:**
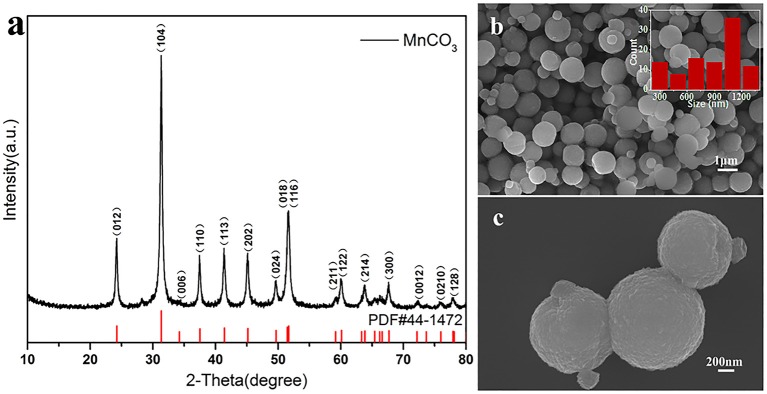
**(a)** XRD pattern of MnCO_3_ and the standard pattern of MnCO_3_ (PDF#44-1472) is shown as reference; **(b,c)** SEM images of MnCO_3_ at different magnification. The inset shows the size distribution of these particles.

The first step is to synthesize MnCO_3_ microspheres successfully, which is a key for the preparation of LiMn_2_O_4_ microspheres. We have successfully prepared the MnCO_3_ microspheres using precipitation. Powder X-ray diffraction analysis of spherical products after the precipitation reaction was employed to identify the crystallographic phase. Figure 2a shows the XRD pattern of the obtained products and the standard pattern of MnCO_3_. All the diffraction peaks can be indexed to the well-crystallized hexagonal MnCO_3_ (JCPDS#44-1472). No other impurities can be observed in the XRD pattern. Figures 2b,c displays the SEM images of spherical MnCO_3_. It is evident that MnCO_3_ spheres are uniform and monodispersed with rough surface with an average diameter of about 1.0 μm. In addition, we found that the morphology of the MnCO_3_ was greatly influenced by the preparation conditions during the precipitation. Therefore, we have studied the effects of the main preparation conditions in the precipitation process of the synthesis of MnCO_3_.

#### The Effects of MnSO_4_ Concentration on the Morphology of MnCO_3_

**Figure 3:**
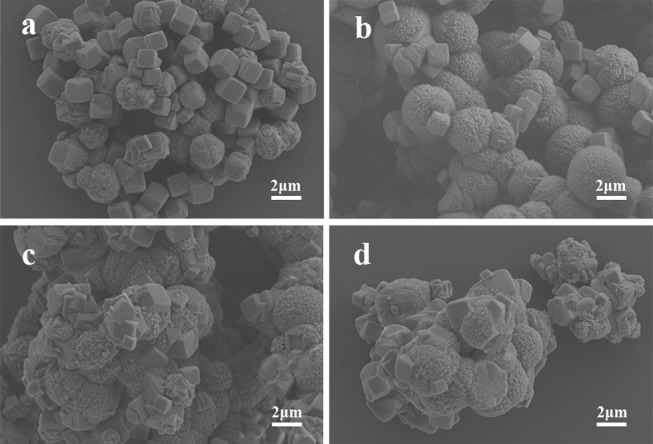
SEM images of MnCO_3_ prepared at different concentrations of MnSO_4_ solution. **(a)** 0.02 M; **(b)** 0.036 M; **(c)** 0.10 M; **(d)** 0.20 M.

The SEM images of MnCO_3_ prepared at the different concentrations of the MnSO_4_ solution are shown in Figure 3. During the preparation, the concentration of NH_4_HCO_3_ was 0.36 M, and other synthesis conditions were maintained. When the concentration of MnSO_4_ solution is 0.02 M, the morphology of MnCO_3_ tends to form more cubes than spheres (Figure 3a). With the increase of the MnSO_4_ concentration to 0.036 M, their morphology gradually transforms from cubical to spherical shapes (Figure 3b). When the concentration of the MnSO_4_ solution is higher than 0.10 M, the morphology of MnCO_3_ becomes an irregular granular shape (Figures 3c,d).

#### The Effects of NH_4_HCO_3_ Concentration on the Morphology of MnCO_3_

**Figure 4:**
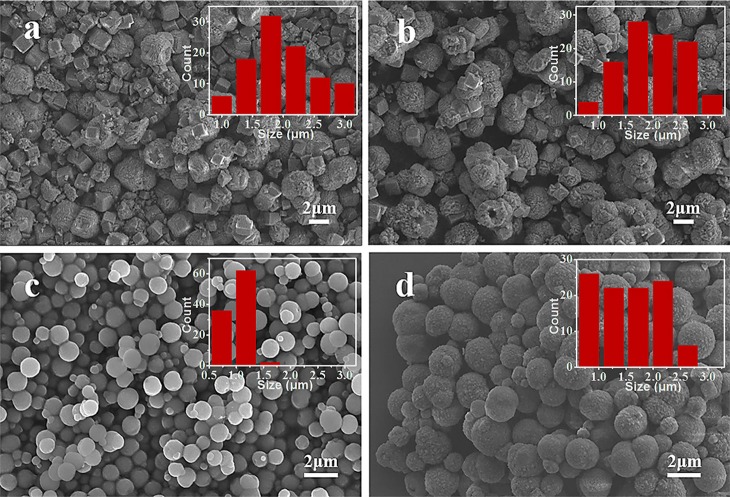
SEM images of MnCO_3_ prepared at different concentrations of NH_4_HCO_3_ solution. **(a)** 0.09 M; **(b)** 0.18 M; **(c)** 0.36 M; **(d)** 0.54 M. The inset shows the size distribution of these particles.

Figure 4 shows the SEM images of MnCO_3_ prepared at different concentrations of the NH_4_HCO_3_ solution. During the preparation, the concentration of the MnCO_3_ solution was 0.036 M, and the other synthesis conditions were maintained. When the NH_4_HCO_3_ concentration is 0.09 M, the morphology of MnCO_3_ is irregular. With the increase of the NH_4_HCO_3_ concentration to 0.18 M, the morphology of MnCO_3_ tends to form spheres. When the concentration of NH_4_HCO_3_ is 0.36 M, the MnCO_3_ spheres own uniform size and good dispersion. The particle size varies from 0.5 to 1.8 μm, mainly in the range of 1.1–1.4 μm, and the average particle size is about 1.0 μm. When the concentration of NH_4_HCO_3_ goes higher as 0.54 M, the spherical MnCO_3_ becomes larger and has a clear agglomeration.

#### The Effects of Solution pH on the Morphology of MnCO_3_

**Figure 5:**
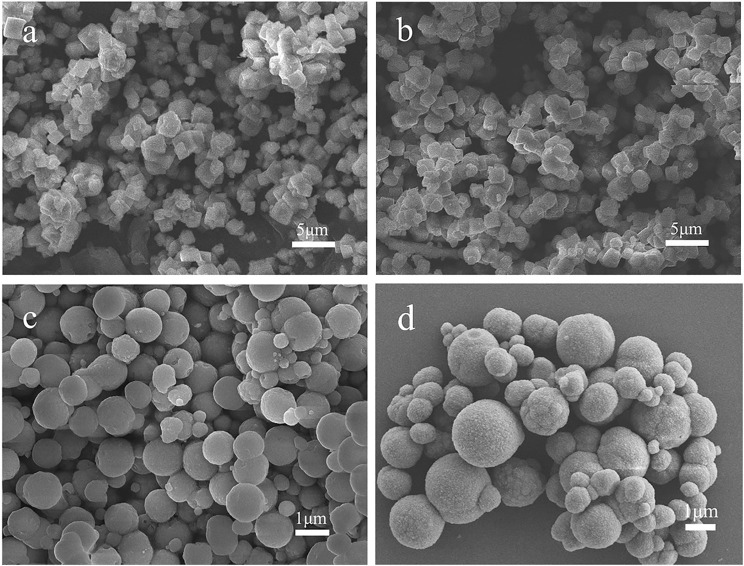
SEM images of MnCO_3_ prepared at different solution pH of 6.5 **(a)**, 7.0 **(b)**, 7.5 **(c)**, and 8.0 **(d)**.

Solution pH proved to be a crucial factor in the controlled formation of the final products (Hong et al., 2003; Kloprogge et al., 2004; Kong et al., 2019; Simon et al., 2019). Herein, the synthesis process in different pH of the above solution was investigated. Figure 5 shows the SEM images of MnCO_3_ prepared at different pH. When the pH is 6.5 and 7.0, MnCO_3_ displays morphologies that are a mixture of cubes and irregular shapes (Figures 5a,b). With the increase of pH value to 7.5, the spherical morphology forms (Figure 5c). When the pH value reaches to 8.0, the size of the microspheres becomes uneven and seriously agglomerated (Figure 5d). This can be explained by the relationship between the pH of the solution and the hydrolysis process of the reactants. An increase in pH can lead to an increase in the hydrolysis rate (Nagao et al., 2004; Hussain et al., 2017). In the reaction process, when the pH value is higher, the hydrolysis of NH_4_HCO_3_ and MnSO_4_ are more complete, which accelerates the formation of MnCO_3_ microspheres, but a hydrolysis speed that is too fast is not conducive to controlling the properties of particles, which will widen the particle size distribution. Instead, the low pH will cause incomplete hydrolysis and irregular appearance of final products.

#### The Effects of Addition Mode on the Morphology of MnCO_3_

**Figure 6:**
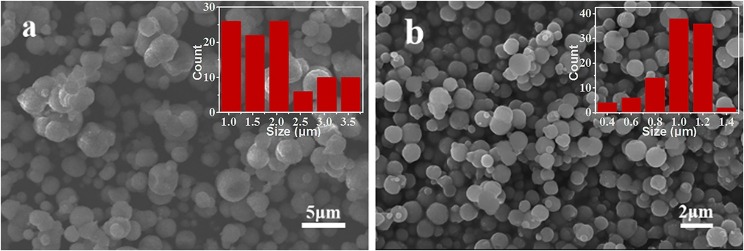
SEM images of MnCO_3_ prepared at different adding mode of NH_4_HCO_3_ solution. **(a)** Added dropwise, **(b)** poured directly. The inset shows the size distribution of these particles.

In addition to the effect of reagent ratio and pH, the addition mode of NH_4_HCO_3_ also has a significant impact on the size and morphology of the synthesized samples. Two synthetic methods—adding the NH_4_HCO_3_ dropwise and pouring it directly into the MnSO_4_ solution—were employed in our experiment. The SEM results of corresponding products are displayed in Figure 6. As shown in Figure 6a, drop-by-drop addition of NH_4_HCO_3_ solution into the MnSO_4_ solution leads to large differences in the size of the MnCO_3_ microspheres, with the average size being 1.95 μm. On the contrary, the size of the MnCO_3_ microspheres is more uniform and the average size reduces to 0.99 μm (Figure 6b) in the later method. This is probably because the nucleation of MnCO_3_ was continuously formed in the solution during the drop-by-drop addition of NH_4_HCO_3_, leading the small microspheres that were formed to be accompanied by the growth of existing microspheres in the solution. In contrast, when the NH_4_HCO_3_ solution was directly poured into the MnSO_4_ solution, the nucleation of MnCO_3_ was formed in a short time and grew up simultaneously. Therefore, the size of the MnCO_3_ microspheres is more uniform.

### The Effects of Heating Temperature and Time on the Morphology of Mn_2_O_3_

**Figure 7:**
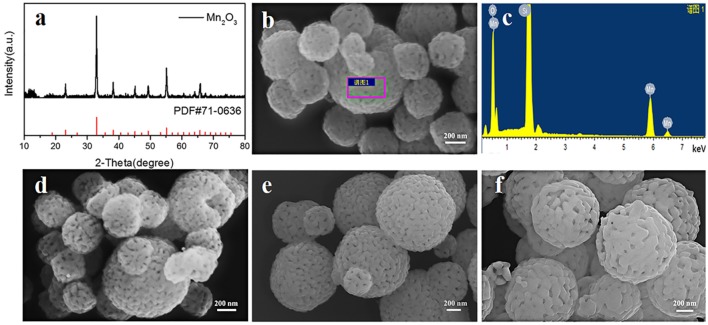
**(a)** XRD pattern of Mn_2_O_3_ heated at 600°C for 5 h, **(b,c)** SEM image and EDS result of Mn_2_O_3_ heated at 600°C for 5 h, **(d–f)** SEM images of Mn_2_O_3_ heated at different temperature and time, **(d)** 600°C, 10 h **(e)** 600°C, 20 h **(f)** 700°C, 10 h.

The second step is to synthesize Mn_2_O_3_ porous microspheres through the decomposition of the MnCO_3_ microspheres by heating treatment. The porous structure of Mn_2_O_3_ microspheres is important for the synthesis of LiMn_2_O_4_ microspheres without structural collapse because this structure can provide more grain shrinkage space during the lithiation process of Mn_2_O_3_ microspheres. We have investigated the influences of the heating temperature and heating time on the morphology of Mn_2_O_3_. The XRD result and SEM images of the corresponding samples are shown in Figure 7. During the decomposition process, the solid phase changes from MnCO_3_ to Mn_2_O_3_ with the release of CO_2_. Reversible oxidation and formation of various manganese oxides occur in the range of 350–560°C (Biernacki and Pokrzywnicki, 1999; Wang et al., 2013), and ultimately, pure phase of Mn_2_O_3_ is formed at above 600°C. Figure 7a shows the XRD pattern of Mn_2_O_3_ heated at 600°C for 5 h. All the peaks are identical to the pure phase of Mn_2_O_3_ (JCPDS#71-0636), which is consistent with the phase transition process in the literature (Wang et al., 2013). On the other hand, a porous microsphere structure consisting of small Mn_2_O_3_ nanocrystals are formed after heating for 5 h (Figure 7b). Furthermore, the EDS result (Figure 7c) also proves that MnCO_3_ has been fully reacted. When the heating time is increased to 10 and 20 h, the spherical shape of the Mn_2_O_3_ porous microspheres is well-maintained, but the sizes of the nanocrystallites increase slightly (Figures 7d,e). As the heating temperature increases to 700°C for 10 h, the nanocrystallites of the Mn_2_O_3_ porous microspheres increase obviously in size and partial microspheres show large holes in the surface (Figure 7f). The above results indicate that both extension of heating time and increase of heating temperature leads the Mn_2_O_3_ crystallites to grow into larger ones by adsorbing surrounding primary particles.

### The Effects of Calcination Temperature, Calcination Time, and Mn_2_O_3_:LiOH Molar Ratio on the Synthesis of LiMn_2_O_4_

**Figure 8:**
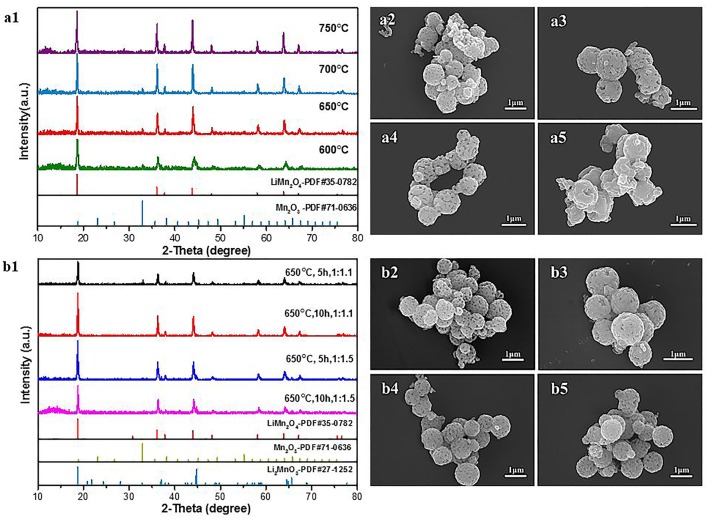
**(a1)** XRD patterns of LiMn_2_O_4_ heated at different temperature. **(a2–a5)** SEM images of LiMn_2_O_4_ heated at different temperature for 5 h. **(a2)** 600°C **(a3)** 650°C **(a4)** 700°C **(a5)** 750°C. **(b1)** XRD patterns of LiMn_2_O_4_ heated at different Mn_2_O_3:_ LiOH and calcination times. **(b2–b5)** SEM images of LiMn_2_O_4_ heated at different Mn_2_O_3_:LiOH and calcination times. **(b2)** 1:1.1, 5 h **(b3)** 1:1.1, 10 h **(b4)** 1:1.5, 5 h **(b5)** 1:1.5, 10 h.

The last step for the synthesis of the LiMn_2_O_4_ spheres is the lithiation reaction of Mn_2_O_3_ porous microspheres with LiOH at high temperature. There are influences of the calcination temperature, calcination time and Mn_2_O_3_:LiOH molar ratio on the synthesis of LiMn_2_O_4_ microspheres. At first, the calcination temperature was adjusted in the range of 600–750°C, and the calcination time and molar ratio of Mn_2_O_3_:LiOH were set as 5 h and 1:1.05, respectively. Figure 8 shows the XRD patterns and SEM images of the corresponding samples. In Figure 8a1, for all the samples, the main characteristic peaks can be indexed as spinel LiMn_2_O_4_ (JCPDS#35-0782), but there is also a small weak peak at 32.9° originating from unreacted Mn_2_O_3_, indicating a slight deficiency in the amount of lithium. It is apparent in Figures 8a3,a4 that the porous spherical structure is well-preserved. It is also clear that LiMn_2_O_4_ spheres are composed of aggregated nanocrystallites with pores existing among them. Compared with the samples heated at 650 and 700°C, a small fraction of broken spheres can be observed in the products under 600°C (Figure 8a2). In Figure 8a5, the samples are piled up by irregular particles, and the holes disappeared. This is due to the structural collapse caused by excessive sintering temperature.

Next, we attempted to increase the molar ratio of Mn_2_O_3_:LiOH to 1.10 and 1.50 to obtain pure spinel LiMn_2_O_4_. Figure 8b1 shows the XRD patterns of the samples synthesized at 650°C with different time. When the molar ratio of Mn_2_O_3_:LiOH reaches 1:1.1 and the calcination time is 5 h, the lithiation reaction is incomplete, remaining as unreacted Mn_2_O_3_. With the extension of calcination time to 10 h, all the diffraction characteristic peaks in the XRD patterns can be identified with standard LiMn_2_O_4_ (JCPDS#35-0782), indicating good crystallinity and high purity for the products. Nevertheless, when the molar ratio of Mn_2_O_3_:LiOH further increases to 1.50, a new characteristic peak appears at 44.8°, which is indexed as Li_2_MnO_3_ (JCPDS#27-1252) phase, indicating that the lithium is excessive in this molar ratio. The above results suggest that the synthesis of pure LiMn_2_O_4_ requires an appropriate range for the molar ratio of Mn_2_O_3_:LiOH, and it is easy to produce byproducts beyond or under the critical values. The morphology and particle size of the products are observed by SEM. As can be seen from Figure 8b2 through Figure 8b5, LiMn_2_O_4_ is composed of uniform microspheres with a rough surface. The diameter of the microspheres is in the range of 0.8–1.0 μm.

### Electrochemical Properties of LiMn_2_O_4_ Microspheres

**Figure 9:**
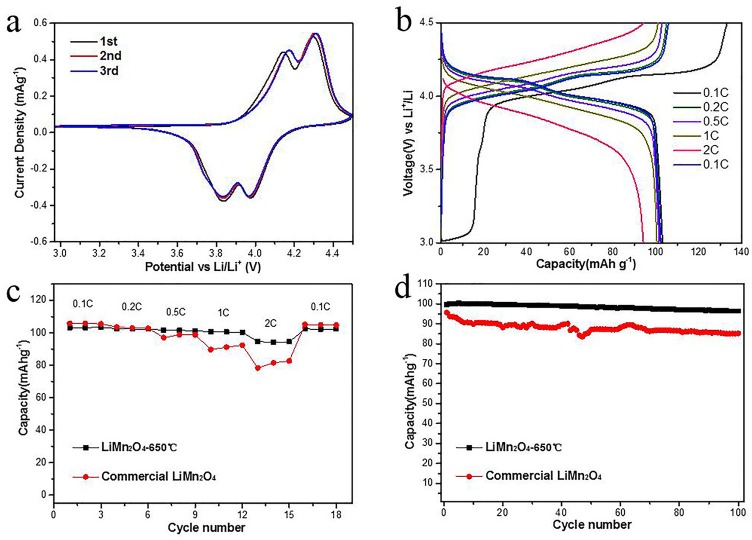
Electrochemical performances of the synthetic porous LiMn_2_O_4_ spheres. **(a)** CV curves at a scan rate of 0.1 mV s ^−1^ in the voltage range of 3.0–4.5 V (vs. Li/Li^+^); **(b)** discharge-charge curves at different rate from 0.1 to 2.0 C **(c)** variation of discharge capacity vs. cycle number of synthetic porous LiMn_2_O_4_ electrodes at 0.1–2.0 C rate; **(d)** cycle performance of synthetic porous LiMn_2_O_4_ at 1.0 C rate.

The electrochemical performance of synthetic porous LiMn_2_O_4_ spheres was discussed as a cathode material for a lithium-ion battery. The cyclic voltammogram (CV) of the synthesized LiMn_2_O_4_ is shown in Figure 9a. Two pairs of separate redox peaks were observed form the CV curves of the synthesized sample, which correspond to the two-step insertion/deinsertion of lithium ion (Thackeray et al., 1983). Figure 9b reveals the current charge/discharge measurement by different rates over a voltage range of 3.0–4.5 V. The discharge capacities at rates of 0.1, 0.2, 0.5, 1.0, and 2.0 C were 103.18, 102.33, 101.50, 100.51, and 94.23 mAh g^−1^, respectively. The rate performance is shown in Figure 9c. As can be seen, the rate performance of LiMn_2_O_4_ synthesized at 650°C is quite good, which demonstrates clearly slower capacity decay with increasing discharge rates. For example, the porous LiMn_2_O_4_ microspheres retain a capacity of 94.23 mAh g^−1^, which is 91.3% of the initial capacity at rate of 0.1 C. This is much higher than that (76%) of the commercial LiMn_2_O_4_ powders at the same rate. When the current went back to a rate of 0.1 C, a capacity of 102.27 mAh g^−1^ was resumed. The cycle stability of LiMn_2_O_4_ synthesized at 650°C at 1.0 C is shown in Figure 9d. The capacity of synthesized LiMn_2_O_4_ remains at 96.42 mAh g^−1^ after 100 cycles and drops by only 3.24% compared to that of the first cycle. For comparison, the commercial LiMn_2_O_4_ exhibits a discharge capacity of 85.15 mAh g^−1^ after 100 cycles, which is much lower than that of the synthesized porous LiMn_2_O_4_ microspheres sample. The good rate and cycling performance of the samples prepared are ascribed to a well-defined structure such as uniform size and high porosity, which is effective in increasing contact area, shortening the transport distance of lithium ions and enhancing the structural stability of electrode material.

## Conclusion

In summary, porous LiMn_2_O_4_ microspheres with an average diameter of about 1 μm have been successfully synthesized by using molten LiOH and porous Mn_2_O_3_ spheres as templates. The morphology and particle size of the products could be conveniently controlled by changing the reactant ratio, pH, adding mode, heating time, etc. The morphology of MnCO_3_ was crucial for the preparation of porous LiMn_2_O_4_ microspheres and was mainly affected by the concentration of reactants and pH value of the solution during the chemical precipitation process. The optimum concentrations of MnSO_4_ and NH_4_HCO_3_ were 0.036 and 0.36 M, respectively, with appropriate pH of 7.5. During the lithiation of Mn_2_O_3_ microspheres, the heating temperature and the ratio between Mn_2_O_3_ and lithium salt were the most significant variables in terms of control over the morphology and purity of the LiMn_2_O_4_ microspheres. Excessive or low temperature would cause the collapse or agglomeration of the prepared LiMn_2_O_4_ microspheres. In addition, unreacted Mn_2_O_3_ can be found in the final products when the amount of lithium salt was deficient. Instead, the byproduct of Li_2_MnO_3_ was easy to generate when there was too much lithium salt. Our work suggested that the uniform porous LiMn_2_O_4_ microspheres were synthesized with the optimum molar ratio of Mn_2_O_3_:LiOH = 1: 1.1 and heated at 650°C for 10 h. Compared with the commercial LiMn_2_O_4_ powder, the synthesized LiMn_2_O_4_ microspheres present better rate capability and cyclability. This work can provide some guidance for the design and synthesis of porous LiMn_2_O_4_ microspheres or other LIBs' electrode materials.

## Data Availability

The raw data supporting the conclusions of this manuscript will be made available by the authors, without undue reservation, to any qualified researcher. Requests to access the datasets should be directed to liuhao1398@cugb.edu.cn.

## Author Contributions

YH and ZZ carried out the experiment and wrote the manuscript. PF and YW participated in the experiment. GL and LM contributed to the discussion. HL and LL supervised the experiment and proofread the manuscript.

### Conflict of Interest Statement

The authors declare that the research was conducted in the absence of any commercial or financial relationships that could be construed as a potential conflict of interest.
